# Characterization of a L-Gulono-1,4-Lactone Oxidase Like Protein in the Floral Nectar of *Mucuna sempervirens*, Fabaceae

**DOI:** 10.3389/fpls.2018.01109

**Published:** 2018-07-30

**Authors:** Hong-Xia Zhou, Richard I. Milne, Xue-Long Ma, Yue-Qin Song, Jian-Yu Fang, Hang Sun, Hong-Guang Zha

**Affiliations:** ^1^College of Life and Environment Sciences, Huangshan University, Huangshan, China; ^2^Institute of Molecular Plant Sciences, University of Edinburgh, Edinburgh, United Kingdom; ^3^Key Laboratory for Plant Diversity and Biogeography of East Asia, Kunming Institute of Botany, Chinese Academy of Sciences, Kunming, China

**Keywords:** L-ascorbate biosynthesis, Floral nectar, L-gulonolactone oxidase like protein, L-galactonolactone dehydrogenase, *Mucuna sempervirens* Hemsl, Nectarin

## Abstract

Floral nectar plays important roles in the interaction between animal-pollinated plants and pollinators. Its components include water, sugars, amino acids, vitamins, and proteins. Growing empirical evidence shows that most of the proteins secreted in nectar (nectarines) are enzymes that can tailor nectar chemistry for their animal mutualists or reduce the growth of microorganisms in nectar. However, to date, the function of many nectarines remains unknown, and very few plant species have had their nectar proteome thoroughly investigated. *Mucuna sempervirens* (Fabaceae) is a perennial woody vine native to China. Nectarines from this species were separated using two-dimensional gel electrophoresis, and analyzed using mass spectrometry. A L-gulonolactone oxidase like protein (MsGulLO) was detected, and the full length cDNA was cloned: it codes for a protein of 573 amino acids with a predicted signal peptide. MsGulLO has high similarity to L-gulonolactone oxidase 5 (AtGulLO5) in *Arabidopsis thaliana*, which was suggested to be involved in the pathway of ascorbate biosynthesis; however, both MsGulLO and AtGulLO5 are divergent from animal L-gulonolactone oxidases. *MsGulLO* was expressed mainly in flowers, and especially in nectary before blooming. However, cloning and gene expression analysis showed that L-galactonolactone dehydrogenase (MsGLDH), a vital enzyme in plant ascorbate biosynthesis, was expressed in all of flowers, roots, stems, and especially leaves. MsGulLO was purified to near homogeneity from raw MS nectar by gel filtration chromatography. The enzyme was determined to be a neutral monomeric protein with an apparent molecular mass of 70 kDa. MsGulLO is not a flavin-containing protein, and has neither L-galactonolactone dehydrogenase activity, nor the L-gulonolactone activity that is usual in animal GulLOs. However, it has weak oxidase activity with the following substrates: L-gulono-1,4-lactone, L -galactono-1,4-lactone, D-gluconic acid-δ-lactone, glucose, and fructose. MsGulLO is suggested to function in hydrogen peroxide generation in nectar but not in plant ascorbate biosynthesis.

## Introduction

L-Ascorbic acid (ascorbate, AsA), is a naturally occurring organic compound belonging to the family of monosaccharides. This compound has antioxidant properties, which help protect against reactive oxygen species (ROS) derived from metabolic activity. AsA also plays an essential role in eukaryotes as an enzyme co-factor in hydroxylation reactions, contributing to diverse processes such as the synthesis of collagen and the demethylation of histones and nucleic acids (Mandl et al., 2009).

L-Ascorbic acid, also known as vitamin C, has multiple applications as a therapeutic for human health, for example in the treatment of common cold, wound healing, and cancer; it is also the vitamin that prevents scurvy. In animals, AsA is synthesized from glucose through intermediates D-glucuronate and L-gulono-1,4-lactone; this is termed the animal pathway. Humans, non-human primates, guinea pigs, bats, and some birds cannot synthesize AsA because L-gulono-1,4-lactone oxidase (GulLO), the terminal enzyme in the biosynthesis process, does not function due to mutation (Chatterjee, 1973). Therefore, these animals including humans need to acquire this vitamin from fresh fruits and green vegetables. Plant-derived AsA is the major source of AsA in the human diet.

L-Ascorbic acid is the most abundant and best characterized water-soluble antioxidant in plants (Foyer and Shigeoka, 2011). Within a plant, AsA mostly accumulates in in photosynthetic organs. The concentration of AsA in cells in green tissues can be up to 5 mM, representing 10% of the total soluble carbohydrate pool (Smirnoff and Wheeler, 2000). As a critical metabolite in plants, AsA has several essential functions in plant physiology, participates in the detoxification of ROS, and has an important role in promoting resistance to senescence and numerous environmental stresses, such as high temperature, dehydration stress, high light, ozone, UV-B radiation, and salt stress. Also, AsA operates as a cofactor, taking part in the regulation of some fundamental cellular processes (e.g., photoprotection, the cell cycle, and cell expansion) and biosynthesis of important plant hormones (e.g., including abscisic acid, jasmonic acid, ethylene, and gibberellic acid) (Smirnoff, 2011; Liang et al., 2017).

The biosynthetic pathways of AsA differ between plants and animal. Plants appear to have multiple pathways for AsA biosynthesis. The primary and most elucidated pathway is the “Wheeler–Smirnoff pathway” which is also called as “D-mannose/L-galactose pathway” or “plant pathway” and start AsA biosynthesis from glucose or mannose (Wheeler et al., 1998, 2015). All genes in this pathway have been identified and at the last step of this pathway, AsA is formed from L-galactono-1,4-lactone in an enzymatic reaction catalyzed by L-galactono-1,4-lactone dehydrogenase (GLDH). Alternative AsA biosynthetic pathways appear to exist in plants, involving galacturonate and glucuronate, but not all enzymes of these pathways have been identified, and little is yet known about their regulation (Bulley and Laing, 2016).

The plant AsA biosynthetic pathway employs GLDH as the terminal enzyme, whereas GulLO has this role in animals. GulLO is deemed to be absent from most of Archaeplastida genomes including higher plants (Wheeler et al., 2015). It is interesting that overexpression of rat GulLO caused an increase in AsA content in tobacco (Jain and Nessler, 2000), potato (Hemavathi et al., 2010), tomato (Lim et al., 2012) and *Arabidopsis* (Lisko et al., 2013). If fed L-gulono-1,4-lactone (L-GulL), detached bean (*Phaseolus vulgaris*) and strawberry (*Fragaria x ananassa*) fruits could convert it to AsA (Baig et al., 1970). GulLO enzyme activity has been detected in hypocotyl homogenates of kidney beans (Siendones et al., 1999), cytosolic and mitochondrial fractions of *Arabidopsis* cell cultures (Davey et al., 1999), and potato tubers (Wolucka and Van Montagu, 2003). An enzyme family exhibiting some similarity to animal GulLO has also been reported in *Arabidopsis* (Maruta et al., 2010). Three putative *Arabidopsis* GulLOs (AtGulLO2, 3, and 5) over-expressed in tobacco BY-2 cell cultures increased AsA after feeding with L-GulL (Maruta et al., 2010). Aboobucker et al. (2017) purified a recombinant *Arabidopsis* GulLO enzyme (AtGulLO5) in a transient expression system. They proved that AtGulLO5 is an exclusive dehydrogenase with an absolute specificity for L-GulL as substrate, thus differing from both existing plant GLDHs and mammalian GulLOs. However, the catalytic efficiency of AtGulLO5 was low.

These findings suggested that there might be an animal AsA biosynthetic pathway analog existing in plants with GulLO as the terminal enzyme. However, up to date, no spontaneous GulLOs had been isolated from plants and no activity from such plant GulLOs has been analyzed. In this study, for the first time, we identified an AtGulLO homolog (named as MsGulLO) in the nectar from *Mucuna sempervirens* Hemsl (Fabaceae), a perennial woody climber bean species that is widely distributed in subtropical regions of China, Bhutan, North East India (West Bengal, Manipur, Sikkim), Japan and Myanmar. We then cloned the full-length cDNA sequence of *MsGulLO* and a L-galactono-1,4-lactone dehydrogenase gene (named as *MsGLDH*) which was used as a reference in the gene expression and phylogenetic analysis. The functional difference between MsGulLO and MsGLDH will be discussed in the context of their phylogenetic relationship and gene expression. MsGulLO was purified to near homogeneity from the nectar using size-exclusion chromatography and the enzymatic activity was assayed *in vitro*. The possible role of MsGulLO in the nectar is also discussed.

## Materials and Methods

### *Mucuna sempervirens* Floral Nectar Collection, pH, Hydrogen Peroxide, AsA, Glutathione, Sugars, and Protein Content Determination

Three *M. sempervirens* (MS) plants grown in greenhouse at Huangshan University (Anhui province, China) were used in this study. Raw nectar was collected from MS flowers in April 2017. Pooled nectar was filtered through 0.22 μm syringe filters (Millipore) to remove dirt and pollen granules from the samples, and stored at -80°C prior to use. The pH of individual nectar samples from 15 flowers was measured by using a pH meter (Model FiveGo F2, Mettler Toledo) with an InLab Micro Probe (Mettler Toledo). Total sugar concentration of MS nectar samples was estimated as the Brix value, obtained with a low-volume hand-held refractometer (Eclipse, Bellingham and Stanley, Tunbridge Wells, United Kingdom). The determination of sugars and L-gulono-1,4-lactone in MS nectar was performed with an EClassical 3100 high-performance liquid chromatograph (Elite, Dalian, China) equipped with a refractive index detector (RI-201H, Shodex, Japan). The separation was performed using a carbohydrate column (SC1011, Shodex, Japan), and purified water was used as an eluent for analysis at a flow rate of 0.8 ml min^−1^ at 85°C. The concentration of hydrogen peroxide in MS nectar samples was measured using a commercially available colorimetric assay kit (Sangon Biotech Co., Ltd., Shanghai, China) according to the manufacturer’s instructions. The concentration of total and reduced AsA in nectar was determined following the method of Kampfenkel et al. (1995). Glutathione (GSH) and oxidized GSH (GSSG) in nectar were measured with a GSH/GSSG Assay Kit (Beyotime Biotech Co., Ltd., Shaghai, China). Protein content in the nectar samples was determined according to the method of Bradford (1976), using bovine serum albumin as the standard.

### Two-Dimensional Gel Electrophoresis (2-DE) and Mass Spectrometry

Because of high protein concentration in MS nectar, filtered nectar was directly used for 2-DE without further concentration. Isoelectrofocusing (IEF) was carried out using a PROTEAN i12 IEF system (Bio-Rad) and a 7-cm Immobiline Dry Strips (linear pH 3−10, Bio-Rad) as described in Ma et al. (2017). Second-dimension electrophoresis was carried out on 12% polyacrylamide gels in a Mini-PROTEAN Tetra system (Bio-Rad) following the manufacturer’s instructions. The 2-DE gels were double stained with Coomassie Brilliant Blue G250 and silver nitrate. Samples were run in triplicate.

For protein identification, spots of interest were manually excised from 2-DE gels and subjected to in-gel digestion using trypsin as the protease, followed by protein identification using a 5800 tandem matrix-assisted laser-desorption ionization time-of-flight mass spectrometer (Applied Biosystems, Foster City, CA, United States). The combined mass spectrometry (MS) and tandem MS (MS/MS) peak lists were analyzed using GPS (Global Proteome Server) Explorer Software 3.6 (Applied Biosystems) with a Mascot search engine (MASCOT version 2.3; Matrix Science, London, United Kingdom), and searched against the National Center for Biotechnology Information database (NCBIprot 20170707). The taxonomic restrictions were set to NCBI-Other green plants. The MS proteomics data have been deposited to the ProteomeXchange Consortium via the PRIDE (Vizcaíno et al., 2016) partner repository with the data set identifier PXD010067.

### Cloning of *MsGulLO* and *MsGLDH*

**FIGURE 1:**
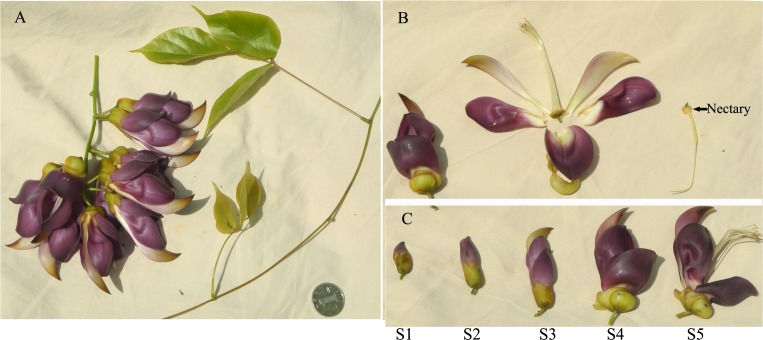
*Mucuna sempervirens* plant. **(A)** Inflorescence, stems and leaves. **(B)** The structure of flower, with nectary indicated by an arrow. **(C)** Different developmental stages of flower. S1 stage, very young flower (corolla less than 1.5 cm long); S2 stage, young flower (corolla ca 2.5 cm long, almost half the length of the adult flower); S3 stage, near adult flower with more than 3 cm long corolla but without enlarged calyx and nectar production; S4 stage, adult flower with nectar in enlarged calyx and keel; S5 stage, old flower with keel opened, and pistil and stamen no longer contained within the keel.

For RACE, total RNA samples were isolated from the stylopodium (which contains the nectary) from MS flowers (stage S4 as shown in **Figure 1**) using a RNeasy Plant Mini kit (Qiagen) with an on-column DNase treatment according to the manufacturer’s protocol. The quality and concentration of extracted RNA was assessed using a Nanodrop Spectrophotometer (ND-2000, Thermo Fisher Scientific).

A protein detected by the above analysis was determined to be an L-gulonolactone oxidase, here named as MsGulLO. To clone the full-length cDNA of *MsGulLO*, a combination of 3′ and 5′ RACE PCR was performed using a SMARTer RACE cDNA Amplification Kit (Clontech) following the manufacturer’s instructions. A primer named as GulLO-F for 3′ RACE was designed according to the well conserved regions in sequence alignments of five GulLO genes from *Cajanus cajan* (accession no. XM020368474), *Glycine max* (accession no. XM003548358), *Lupinus angustifolius* (accession no. XM019600852), *Medicago truncatula* (accession no. XM013589106), and *P. vulgaris* (accession no. XM007135246).

To clone the reference MS *GLDH* cDNA (here named as *MsGLDH*), a primer named as GLDH-F was designed for 3′ RACE according to the conserved motif sequences of five GLDH genes from *Glycine max* (accession no. NM001249443), *Lupinus angustifolius* (accession no. XM019572875), *Medicago truncatula* (accession no. XM003590185), *P. vulgaris* (accession no. XM007145487), and *Vigna angularis* (accession no. XM017559956). The primers for 5′ RACE were then designed according to the sequence data from 3′ RACE for both *GULO* and *GLDH*. Products of RACE reactions were directly sequenced without any cloning steps. The resulting sequence reads were assembled to generate the full length cDNA sequences of *MsGulLO* and *MsGLDH*. Primer sequences used are detailed in **Supplementary Table S1**. The full length sequences of *MsGulLO* and *MsGLDH* were deposited in GenBank.

### Analysis of Gene Expression

To investigate the relative expression of *MsGulLO* and *MsGLDH* transcripts in different plant organs, total RNA samples were extracted from petals, calyx, and stamens of flowers, all at developmental stage S4 (**Figure 1**), and from stems, leaves, and roots. RNA was also extracted from stylopodia containing nectaries from flowers at five different developmental stages (S1, S2, S3, S4, and S5; **Figure 1**). MS flowers secrete nectar from stage 4 until they are pollinated (Zha HG; personal observation). cDNA synthesis was performed according to the manual using the Transcriptor First Strand cDNA Synthesis Kit (Roche Diagnostics) with oligo(dT) primer. Quantitive PCR was performed with the LightCycler 96 system (Roche Applied Science) and FastStart Essential DNA Green Master (Roche Diagnostics). The PCR conditions were as follows: 94°C for 5 min and then 45 cycles of PCR (95°C for 15 s, 53°C for 15 s and 72°C for 15 s). Gene-specific primers (GulLORTF and GulLORTR for *MsGULLO*; GLDHRTF and GLDHRTR for *MsGLDH*), designed according to the cloned full-length sequences, are listed in **Supplementary Table S1**. The abundance of transcripts was analyzed using the delta delta Ct (ddCt) method based on relative quantification with normalizing to the housekeeping gene: *18S rRNA*. The bean *18S rRNA* specific primers, 18SF and 18SR, were used for the amplification. We also tested the *EF-1α* and *actin* genes to be used as reference genes in qPCR, and all showed similar expression patterns. All qPCR experiments were repeated with three independent biological replicates and amplicon specificity was checked by high-resolution melting curve analysis.

### Bioinformatics Analyses

The theoretical isoelectric point (pI), molecular weight, and hydrophobicity of MsGulLO were calculated using the ProtParam tool available through the ExPasy Web site^1^ (Gasteiger et al., 2005). Protein domains were predicted using the Pfam database^2^ (Finn et al., 2016). N-terminal signal peptide and cleavage sites were predicted using SignalP 4.1 server^3^ (Nielsen, 2017). Predictions of MsGulLO and MsGLDH subcellular localizations were performed by the TargetP webserver^4^ (Emanuelsson et al., 2007) and YLoc^5^ based on the “YLoc-HighRes Plants model” (Briesemeister et al., 2010).

### Phylogenetic Analysis

Twenty six terminal enzymes in the biosynthesis of AsA or its analogs, including both animals and plant sources, were retrieved from Swiss-Prot and used for phylogenetic analysis, including MsGulLO, MsGLDH, plant GulLOs, plant GLDHs, animal GulLOs, ALOs, GulLDH, GalLO, and GUO. Molecular Evolutionary Genetics Analysis (MEGA) software version 7.0 was used for the construction of sequence alignments and phylogenetic trees with the amino acid sequences (Kumar et al., 2016). Evolutionary trees were inferred using Maximum Likelihood method. The LG (Le and Gascuel, 2008) model, with invariant sites and gamma distribution (LG + I + G) was estimated as the best-fitting model of amino acid substitution from the data. Bootstrap values were calculated using 1000 replications.

### MsGulLO Purification and Enzymatic Assays

MsGulLO was part-purified from raw MS nectar using SEC. Briefly, 20 ml pooled MS floral nectar was used for MsGulLO purification. The proteins in the nectar were concentrated 10 times by ultracentrifugal filtering with Amicon Ultra centrifugal filters (cut-off 10 kDa; Millipore). Two milliliter of the concentrate was then applied onto a Superdex 75 column (60 cm × 1.6 cm) equilibrated in 100 mM sodium acetate buffer, pH 5.0. The column was run at a flow rate of 30 ml h^−1^, and 1 ml fractions were collected. Protein elution was monitored by A280. Peak fractions were analyzed by SDS-PAGE under non-reducing conditions, and fractions (fraction 26-28; **Supplementary Figure S1**) containing MsGulLO protein of sufficient purity were pooled, and quantified. The isolated protein was run on a denaturing SDS polyacrylamide gel and subjected to mass spectrometry (MALDI-TOF/TOF) to ascertain its purity, then stored at −80°C until further use.

Raw MS nectar and isolated MsGulLO’s L-galactono-1,4-lactone dehydrogenase activity, were each tested for their ability to reduce cytochrome C at 550 nm, following Aboobucker et al. (2017). The degree of L-gulono-1,4-lactone oxidase activity in MsGulLO was measured by monitoring AsA production in the reaction using L-GulL as the substrate, following Aboobucker et al. (2017). MsGulLO’s oxidase activity was assayed spectrophotometrically using an o-dianisidine-peroxidase coupled assay with L-GulL, L-GalL, D-GluL, glucose, fructose, mannose, sucrose, xylose, arabinose, and AsA as substrates according to Bergmeyer (1974). All assays were performed in triplicates, and the mean ± standard deviations are presented.

## Results

### *Mucuna sempervirens* Floral Nectar Contains AsA and Hydrogen Peroxide, but Not L-Gulono-1,4-Lactone

*Mucuna sempervirens* secretes nectar from flower developmental stage S4 (**Figure 1C**) until pollinated; in total ca. 50∼150 μl of nectar per flower. MS nectar was acidic with a pH value of 5.3 ± 0.2 and a total sugar concentration of 25.0 ± 5.2 Brix°(mean ± SD, *n* = 15). HPLC showed that MS nectar in this study was a sucrose rich type, containing sucrose, glucose, and fructose at a ratio of 1: 0.22: 0.32. L-gulono-1,4-lactone was not detected in MS nectar by HPLC using refractive index detection. The concentration of hydrogen peroxide detected in MS nectar was 62.1 ± 10.5 μM (mean ± SD, *n* = 15). The concentration of total and reduced AsA in MS nectar were 4.3 ± 0.5 and 2.4 ± 0.4 μM (mean ± SD, *n* = 6), respectively. Only oxidized GSH (GSSG) was detected in MS nectar, and this had a concentration of 0.74 ± 0.07 μM (mean ± SD, *n* = 6). The mean concentration of protein in MS nectar was 370 μg ml^−1^ (*n* = 15) which was almost ten times higher than reported *Canavalia gladiata* and *Nicotiana tabacum* nectar protein concentration and didn’t need to be concentrated before used for gel electrophoresis analysis (Zha et al., 2012; Liu et al., 2013; Ma et al., 2017).

### A Plant L-Gulonolactone Oxidase Homolog Was Detected in MS Nectar

**FIGURE 2:**
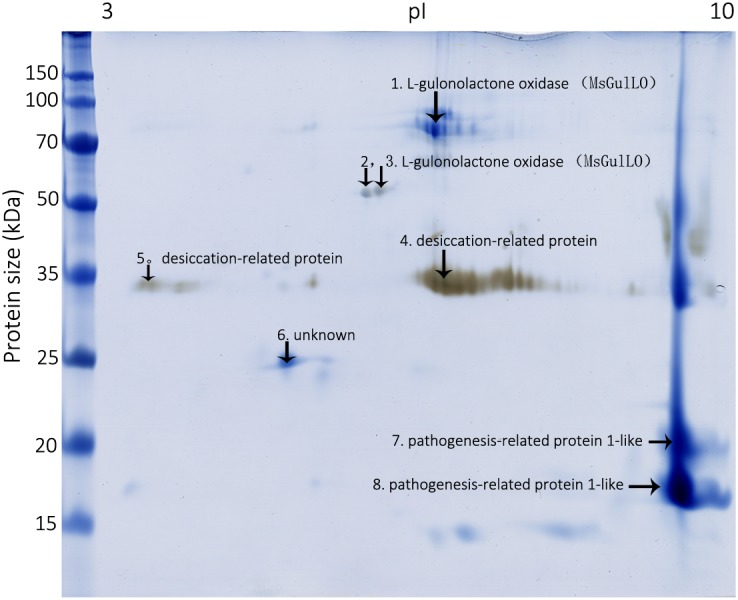
*Mucuna sempervirens* nectarines on 2D gel. Identified nectar proteins on the gel, including MsGulLO, are indicated by arrows.

To identify MS nectar proteins, we performed 2D gel electrophoresis of MS nectar, which yielded more than 10 spots in the gel after visualization by Coomassie Brilliant Blue G-250 and silver staining (**Figure 2**). As we previously reported, most of the MS nectar proteins were alkaline, and ranged in molecular mass from 17 to 100 kDa (Zha et al., 2013). All visible protein spots (19 in total) were subjected to tryptic digestion and then analyzed by MALDI-TOF/TOF. Because proteins might be truncated or modified during the process of 2-DE, different spots in the gel could represent the same protein. In this study, three proteins were successfully identified by mass spectrometry (MALDI TOF/TOF): L-gulonolactone oxidase, a desiccation-related protein, and a pathogenesis-related protein 1-like protein (**Figure 2** and **Supplementary Table S2**). From the first three spots, two peptides, QEDAIDFDITYYR (MW 1648.67) and LYEDIIEEVEQLGIFK (MW 1937.91), were identified; these matched the identity of L-gulonolactone oxidases from *Glycine max* (accession no. XP_006604910), *Cajanus cajan* (accession no. XP_020213315), and *Malus domestic* (accession no. XP_008353193). Therefore, the protein identified as an L-gulonolactone oxidase in MS nectar was designated as MsGulLO.

### *MsGulLO* cDNA Cloning and Amino Acid Sequence Analysis

Using a combination of 5′ and 3′RACE methods, full-length cDNAs encoding MsGulLO and MsGLDH were cloned (accession numbers MF327592 and MG021324, respectively). The *MsGulLO* gene consists of 1722 bp, and encodes a protein of 573 amino acids. In a BLAST search, this gene showed high identity with reported plant GulLOs, with the highest identity (90%) with the sequence of *Glycine max* L-gulonolactone oxidase-4 (accession no. XM_006604847). However, the closest match with a well functional characterized animal GulLO (accession no. P10867 from *Rattus norvegicus*) was only 27%. The full amino acid sequence of MsGulLO was deduced from the cDNA sequence, and subjected to a Pfam search which revealed an ALO family domain at positions 375−520 (*E*-value: 5.6e^−13^) and an FAD binding domain at positions 2−133 (*E*-value: 6.3e^−22^). However, in common with other plant GulLOs, MsGulLO doesn’t have a FAD-binding motif in its N terminus (Leferink et al., 2008; Aboobucker and Lorence, 2016). The mature MsGulLO protein had a neutral isoelectric point of 6.72, and a predicted molecular mass of 61,962.52 Da which is consistent with the 2-DE results (**Figure 2**). The first 18 amino acids of MsGulLO were predicted to form a signal peptide by SignalP, which suggested that MsGulLO is a secreted protein. In addition, TargetP and YLoc predicted that MsGulLO would enter the secretory pathway, and become located in extracellular space. Mature MsGulLO is predicted to be a stable and hydrophilic protein, with an instability index (II) of 30.23, and a grand average of hydropathicity (GRAVY) score of −0.315. These predictions are in agreement with the hypothesis that MsGulLO is secreted out from the nectary, and presents as a soluble protein in nectar.

The mass spectrometric data of MsGulLO confirmed the presence of 17 peptides that matched the predicted masses derived from the translated sequence of the *MsGulLO* gene (**Supplementary Table S3**). These peptides covered 36.8% of the total amino acid sequence of the mature MsGulLO protein. Thus, we conclude that the *MsGulLO* gene encodes a plant L-gulono-1,4-lactone oxidase homolog, MsGulLO.

The *MsGLDH* gene consists of 1752 bp, and encodes a protein of 583 amino acids. The sequence of the *MsGLDH* gene showed high identity with reported plant GLDHs, with the highest identity (93%) being to the sequence of *Cajanus cajan* L-galactono-1,4-lactone dehydrogenase (accession no. XM_020367752). The MsGLDH protein was alkaline (theoretical pI: 8.46) and had a predicted molecular mass of 66,171.67 Da. Two ALO family domains at positions 255−343 and 370–576 (*E*-value: 8.5e^−06^ and 8.8e^−12^), and an FAD binding domain at positions 4−135 (*E*-value: 2.5e^−28^), were detected in this MsGLDH protein sequence by Pfam search. However, unlike MsGulLO, MsGLDH was predicted to be a mitochondrial protein, containing no signal peptide but a FAD-binding motif in the N terminus. This prediction is in agreement with plant GLDHs being localized in the mitochondria (Aboobucker and Lorence, 2016). MsGLDH had an instability index (II) of 44.97, which indicated that it is not theoretically stable.

### MsGulLO and Other Plant GulLOs Are Divergent From Other Aldonolactone Oxidoreductases

**FIGURE 3:**
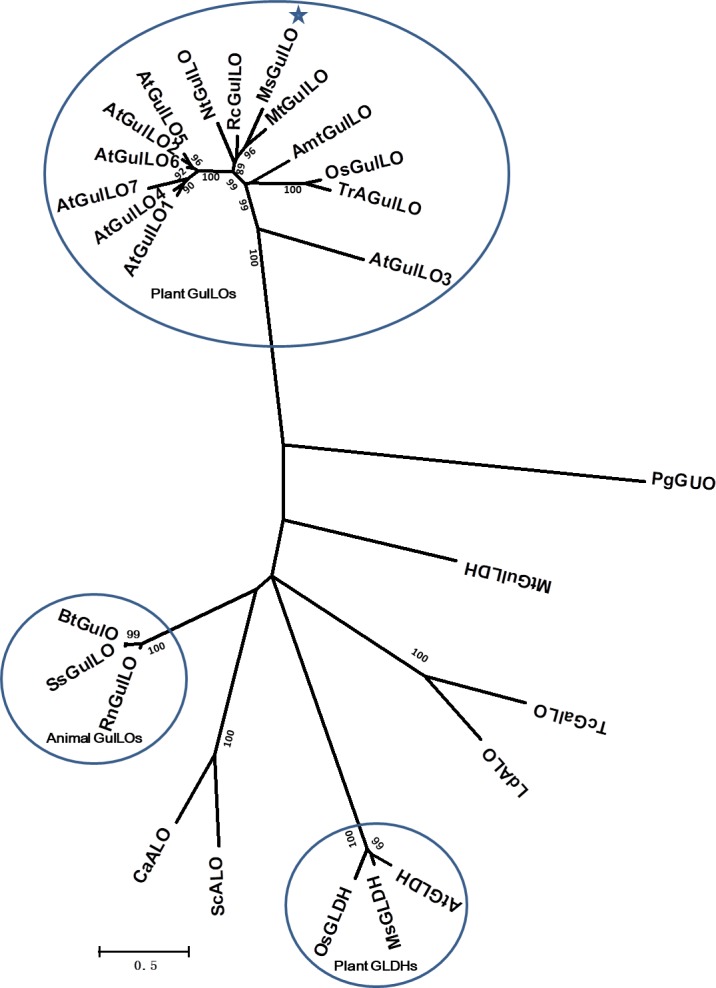
Phylogenetic analysis of 26 terminal enzymes that are involved in the biosynthesis of AsA or its analogs. The phylogenetic relationship was reconstructed by using the Maximum Likelihood method and the Le and Gascuel (2008) model by Mega 7.0. MsGulLO is indicated by a star. Plant GulLOs, plant GLDHs, and animal GulLOs are circled. Numbers on branches indicate the bootstrap percentage values (>80%) calculated from 1000 bootstrap replicates. The species used in the unrooted phylogeny tree construction are as follows, with names followed by accession number. GulLOs from plants: *Amborella trichopoda* (AmtGulLO; W1PKS5), *Arabidopsis thaliana* (AtGulO1 to AtGulO7; Q9C614, Q6NQ66, Q9LYD8, Q9FM82, O81030, O81032, and Q9FM84), *Medicago truncatula* (MtGulLO; A0A072TYF3), *Mucuna sempervirens* (MsGulLO; A0A290U7F5), *Nicotiana tabacum* (NtGulLO; A0A1S4B1A6), *Oryza sativa* (OsGulLO; Q10I64), *Ricinus communis* (RcGulLO; B9SVF9), *Triticum aestivum* (TrAGulLO; A0A077RZP9); GulLOs from animals: *Bos taurus* (BtGulLO; Q3ZC33), *Rattus norvegicus* (RnGulLO; P10867), *Sus scrofa* (SsGulO; Q8HXW0); GLDHs from *Arabidopsis thaliana* (AtGLDH; Q9SU56), *Mucuna sempervirens* (MsGLDH; AVM41577), *Oryza sativa* (OsGLDH; Q2QXY1); ALOs from *Candida albicans* (CaALO; O93852), *Leishmania donovani* (LdALO; C8CCV9), *Saccharomyces cerevisiae* (ScALO; P54783). GulLDH from *Mycobacterium tuberculosis* (MtGulLDH; P9WIT3). GalLO from *Trypanosoma cruzi* (TcGalLO; Q4DPZ5). GUO from *Penicillium griseoroseum* (PgGUO; Q671X8).

From the protein sequences available, an unrooted maximum likelihood phylogenetic tree was produced (**Figure 3**). It showed that those aldonolactone oxidoreductases that function as terminal enzymes in the biosynthesis of AsA in different organisms formed two distantly related clades. MsGulLO was grouped with other plant GulLOs with strong support values. Plant GLDHs, animal GulLOs, ALOs, GulLDH, GalLO, and GUO from other organisms formed another clade. Based on this, we speculated that so-called plant GulLOs have functions that are distinct from animal GulLOs and other well-established terminal enzymes in AsA biosynthesis. All seven GulLOs from *Arabidopsis thaliana* (AtGulLOs) were incorporated in this analysis and MsGulLO was closely related to the clade formed by AtGulLO 2, 5, and 6. Transgenic analysis suggested that AtGulLO 5 played roles in AsA biosynthesis (Maruta et al., 2010; Aboobucker et al., 2017), which indicates that MsGulLO probably has a similar function.

### *MsGulLO* Is Mainly Expressed in the MS Nectary

**FIGURE 4:**
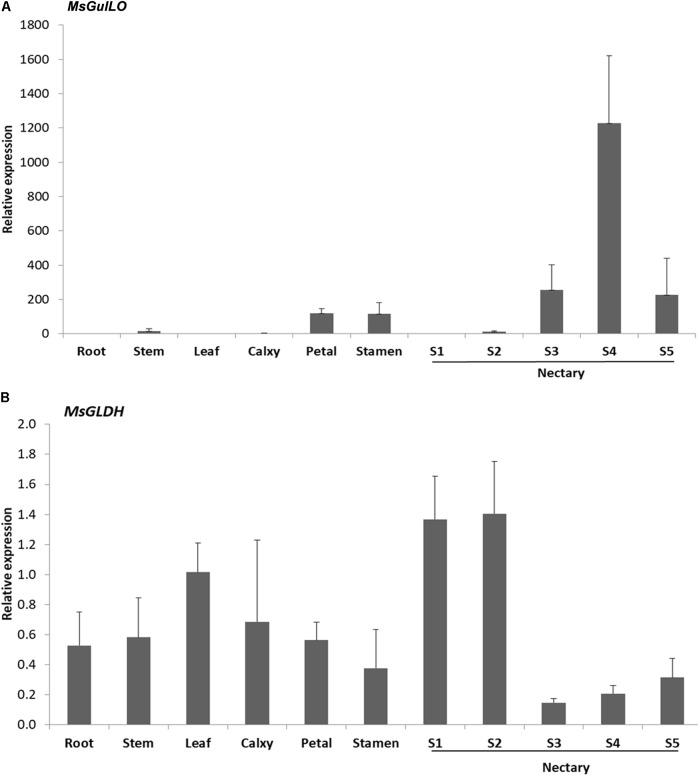
Determination by qRT-PCR of transcript levels of *MsGulLO and MsGLDH* in various tissues. **(A)** *MsGulLO* expression patterns. **(B)** *MsGLDH* expression patterns. Whole RNA was isolated from the following tissues: stems, roots, petals, calyxes, stamens, leaves; S1−S5, nectaries at developmental stage 1−5. All data were normalized to 18S rRNA transcript levels. Values represent mean ± SE from three biological repeats.

Expression analysis of the *MsGulLO* gene in MS leaf, stem, root, petal, stamen, and nectaries at five developmental stages was accomplished by qRT–PCR. The relative expression level of *MsGulLO* was high in nectaries at developmental stage 3, 4, and 5 (**Figure 4A**). *MsGulLO* transcripts were also detected in petal, stamen, and nectary at developmental stage 2, and in much lower quantities in the stem; they were not detected in the root, leaf, petal, or nectary at developmental stage 1. Our results indicate that *MsGulLO* is mainly expressed in flowers, and especially in the nectary. *MsGulLO* gene transcripts start to accumulate in the nectary ahead of blooming, and before nectar secretion, which demonstrates that it is synthesized before these things happen.

However, *MsGLDH* showed a completely different expression pattern to *MsGulLO* (**Figure 4B**). *MsGLDH* transcripts were detected in all the tissues tested in this study. Unlike MsGulLO, the relative expression level of MsGLDH was low in nectaries at developmental stage 3, 4, and 5, but high in developmental stage 1 and 2 (**Figure 4B**). Therefore, *MsGLDH* is shown to be constitutively expressed and its function might not be related with nectary or flower development. It is consistent with this that no MsGLDH was detected in MS nectar in this study.

### MsGulLO Had No L-Gulono-1,4-Lactone Oxidase or L-Gulono-1,4-Lactone Dehydrogenase Activity in AsA Biosynthesis

**FIGURE 5:**
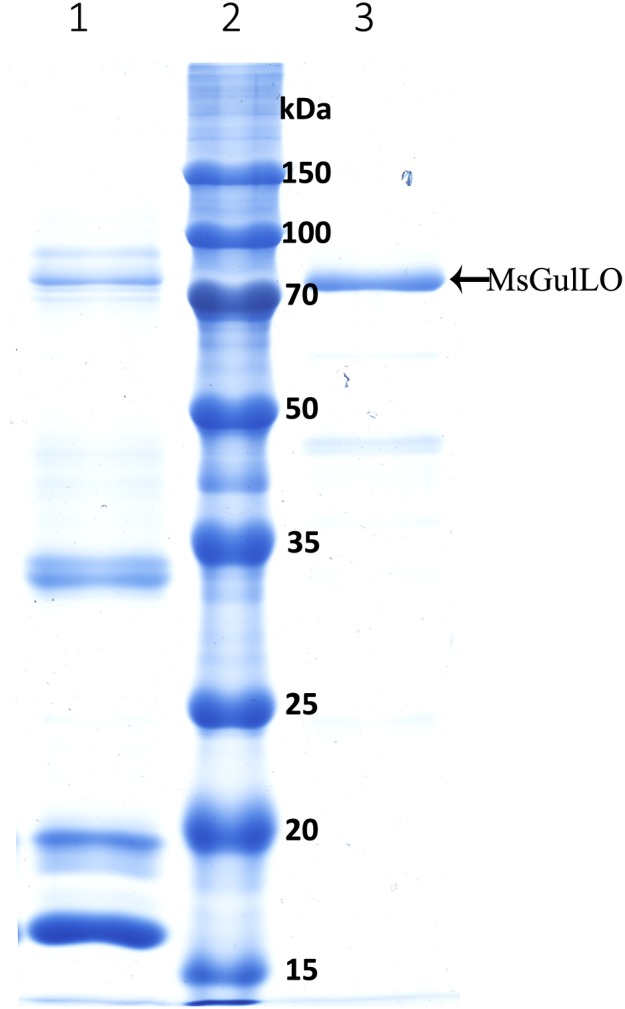
SDS–PAGE of *M. sempervirens* nectar proteins and isolated MsGulLO. Lane 1, MS nectar proteins; lane 2 molecular weight markers; lane 3, Isolated MsGulLO.

In this study, MsGulLO was isolated from MS nectar proteins and other nectar components, such as sugars, using SEC, and the elution profile of MS nectar proteins is depicted in **Supplementary Figure S1**. MsGulLO containing fractions (no. 26−28) were pooled for subsequent analysis (**Supplementary Figure S1**). The isolated MsGulLO migrated as one major band in SDS-PAGE gel with a MW of 70 kDa with several very weak bands (**Figure 5**). The mass spectrometric peptide fingerprinting analysis proved that the part-purified protein was identical to the MsGulLO protein identified from 2-DE gel (data not shown).

Both raw MS nectar and isolated MsGulLO showed no GLDH and GulLO activity as animal GulLOs, and no AsA was generated during the assay; this is consistent with purified recombinant AtGulLO5 having no GulLO activity (Aboobucker et al., 2017). AtGulLO5 was demonstrated to have GLDH activity (Aboobucker et al., 2017), but this study showed no GLDH activity for MsGulLO. Using an o-dianisidine-peroxidase coupled assay, MsGulLO showed weak oxidase activity toward L-GulL (0.08 ± 0.02 units mg^−1^), L-GalL (0.10 ± 0.02), D-GluL (0.08 ± 0.02), glucose (0.08 ± 0.02), and fructose (0.06 ± 0.01) (mean ± SD, *n* = 3 in each case). L-GulL, L-GalL, and D-GluL were not detected in MS nectar by using HPLC with refractive index detection. MsGulLO showed no oxidase activity to raffinose, mannose, sucrose, arabinose, and AsA even though both sucrose and AsA were present in MS nectar. Therefore, both glucose and fructose are probably MsGulLO’s natural substrates.

## Discussion

AsA (vitamin C) is an enzyme co-factor in eukaryotes that also plays an important role in protecting photosynthetic eukaryotes against damaging ROS derived from the chloroplast (Wheeler et al., 2015). In many animal lineages, L-gulonolactone oxidase (GulLO) is the terminal enzyme in the AsA biosynthetic “animal” pathway. Growing molecular and biochemical evidence from photosynthetic eukaryote lineages has demonstrated an alternative “plant” pathway, also called the “Smirnoff-Wheeler pathway,” in which GulLO is functionally replaced with GLDH (Wheeler et al., 2015). Another AsA biosynthetic pathway existing in plants has been suggested in which the oxidation of L-GulL to AsA is the final step, and GulLO is the terminal enzyme (Maruta et al., 2010; Aboobucker et al., 2017). An enzyme family in plants, which includes AtGulLO1∼7 from *A. thaliana*, was reported to exhibit some similarity to animal GulLO (Maruta et al., 2010). Therefore, detailed characterization of the plant GulLOs is important, but it remains very rare. Maruta et al. (2010) reported that overexpression of AtGulLO 2, 3, or 5 in tobacco cell lines could result in increased AsA levels after L-GuL feeding. However, they failed to obtain the recombinant protein and test the enzymatic activity directly. Until recently, the recombinant AtGulLO5 was firstly isolated and characterized *in vitro* and demonstrated to be not an oxidase but a dehydrogenase which could convert L-GulL to AsA with an absolute specificity for L-GulL (Aboobucker et al., 2017). This investigation also demonstrated that AtGulLO5 is different from the existing plant GLDHs (specific to L-GalL) or mammalian GulLOs.

Here we describe the characterization of an AtGulLO5 homolog, MsGulLO, from the legume *Mucuna sempervirens* (MS), achieved by direct protein purification, enzymatic assays, gene cloning, and expression analysis. Our data did not support the hypothesis that MsGulLO and its plant homologs are the terminal enzyme in the suggested alternative plant AsA biosynthetic pathway. First, no L-gulonolactone oxidase or dehydrogenase activity was detected. Adding L-GulL into MS nectar or isolated MsGulLO did not result in any detectable AsA generation, which likewise indicates that MsGulLO can’t convert L-GulL to AsA (data not shown). In addition, no L-GulL could be detected in MS nectar. Secondly, it is known that flavin plays essential roles in both animal GulLO and plant GLDH activity (Smirnoff, 2001). However, MsGulLO is not a flavin-containing protein, and no flavin could be detected in MS nectar by fluorescence analysis. The presence or absence of FAD from the system had no effect on MsGulLO’s oxidase activity (data not shown). The FAD-binding motif is not present in the protein sequence of MsGulLO, AtGulLOs, or other so-called plant GulLOs (Leferink et al., 2008; Aboobucker and Lorence, 2016). This indicated that plant GulLOs might have different activity and/or a distinct catalysis mechanism from animal GulLOs. Thirdly, all plant GulLOs including MsGulLO and AtGulLOs were predicted to be secretory proteins with a predicted signal peptide. Our finding confirmed this prediction because MsGulLO is secreted into nectar and mainly expressed in the flower and nectary. However, animal GulLOs and plant GLDHs are not secretory proteins (Wheeler et al., 2015). Plant GLDHs are located in mitochondria, and have a different destination from plant GulLOs. This also suggests that Plant GLDHs and GulLOs carry out different functions in plants. Fourthly, even though plant GulLOs share high sequence similarity with each other, the identity between plant GulLOs and animal GulLOs is very low, less than 30% (Aboobucker and Lorence, 2016). Phylogenetic analysis also showed that plant GLDHs are far closer to animal GulLOs than either are to plant GulLOs, which indicates that plant GulLOs probably have a different evolutionary origin to either, and perform a different physiological function. Fifthly, AsA is mostly produced and accumulates in photosynthetic organs in land plants, such as leaves (Gest et al., 2013). However, in this study, no *MsGulLO* transcripts were detected in leaves. Therefore, MsGulLO looks unlikely to be involved in AsA biosynthesis.

The true function of MsGulLO in MS nectar remains unclear. Isolated MsGulLO did show a weak glucose and fructose oxidase activity, which could produce hydrogen peroxide using glucose and fructose as the substrate. High concentrations of hydrogen peroxide in nectar has been deemed to protect the nectary from microorganism growth (Carter and Thornburg, 2004a; Nocentini et al., 2015; Roy et al., 2017). To our knowledge, nectarin V (NEC5) from tobacco plants is the only protein exhibiting glucose oxidase activity that has been identified in nectar, and NEC5 is a flavin-containing berberine bridge enzyme-like protein (Carter and Thornburg, 2004b). With the high concentration of simple sugars present in tobacco nectar, the likely function of NEC5 was to generate the antimicrobial levels of hydrogen peroxide found therein; it hence plays an important role in the “nectar redox cycle”(Carter and Thornburg, 2004a). Both MsGulLO and NEC5 have glucose oxidase activity, but MsGulLO didn’t require FAD for its oxidase activity. We determined that glucose, fructose and hydrogen peroxide all coexist in MS nectar even though the concentration of hydrogen peroxide was not as high as in reported tobacco nectar (Carter and Thornburg, 2004a). Because the concentration of AsA was ten times less than that of hydrogen peroxide in MS nectar, it is doubtful that AsA could detoxify hydrogen peroxide in the nectar. We also noticed that the concentration of hydrogen peroxide in individual MS nectar samples from different flowers could vary dramatically. It looks like that the generation of hydrogen peroxide in nectar might be triggered by some external stimulus, such as microorganisms introduced by pollinators or wind, and that it is under rapid regulation. In addition, MsGulLO has no ascorbate peroxidase activity, and we found that it could not produce hydrogen peroxide using AsA as the substrate (data not shown). Thus, we suggest that MsGulLO might function in the generation of hydrogen peroxide in nectar using glucose and fructose as substrate. However, the mechanism regulating hydrogen peroxide metabolism in nectar is still unknown, and hence requires further investigations. Even though AsA was detected in MS nectar, we couldn’t find any evidence to link MsGulLO’s activity with the generation of AsA.

## Conclusion

In this study, an L-gulonolactone oxidase like protein (MsGulLO) was identified in the floral nectar from MS (Fabaceae) by 2-DE and mass spectrometry. The full length *MsGulLO* cDNA was cloned, and found to encode a protein of 573 amino acids with a predicted signal peptide; it was hence predicted to enter the secretory pathway. MsGulLO has high similarity to other plant GulLOs, such as AtGulLO5 in *A. thaliana* which was suggested to be involved in the pathway of L-AsA biosynthesis. Phylogenetic analysis shows that MsGulLO and plant GulLOs are divergent from animal L-gulonolactone oxidases, whose functions are well characterized. MsGulLO was a secreted protein and expressed only in flowers and especially in nectary before blooming. However, cloning and gene expression analysis showed that MsGLDH, a validated vital enzyme in plant AsA biosynthesis, was located in the mitochondria, whereas it is expressed in flowers, roots, stems, and especially leaves. MsGulLO was purified to near homogeneity from raw MS nectar by gel filtration chromatography. The enzyme was determined to be a neutral monomeric protein with an apparent molecular mass of 70 kDa. MsGulLO is not a flavin-containing protein and has no regular GulLO or GLDH activity, however, it does have oxidase activity using glucose and fructose as natural substrates. MsGulLO is suggested to function in maintaining the homeostasis of hydrogen peroxide in nectar but to not be involved with AsA biosynthesis in nectar or other tissues in the plant.

## Author Contributions

H-GZ, H-XZ, and HS conceived and designed the research. X-LM, Y-QS, and H-XZ conducted the experiments. J-YF and RM analyzed the data. RM and H-XZ wrote the manuscript.

## Conflict of Interest Statement

The authors declare that the research was conducted in the absence of any commercial or financial relationships that could be construed as a potential conflict of interest.

## Footnotes

1 http://web.expasy.org/protparam/

2 http://pfam.xfam.org

3 http://www.cbs.dtu.dk/services/SignalP/

4 http://www.cbs.dtu.dk/services/TargetP/

5 http://abi.inf.uni-tuebingen.de/Services/YLoc/

## Supplementary Material

FIGURE S1 Elution profile of MsGulLO on a Superdex-75 size exclusion chromatography column.

TABLE S1 Primers used for *MsGulLO* and *MsGLDH* cDNA cloning and qPCR.

TABLE S2 *Mucuna sempervirens* nectar proteins identified by 2-DE and mass spectrometry.

TABLE S3 MsGulLO peptides identified by mass spectrometry (MALDI-TOF/TOF).

## Abbreviations

ALO: D-arabinono-1,4-lactone oxidase
AsA: L-ascorbic acid
D-GluL: D-gluconic acid-δ-lactone
FAD: flavin adenine dinucleotide
GalLO: L-galactono-1,4-lactone oxidase
GlcUR: D-glucuronate reductase
GLDH: L-galactono-1,4-lactone dehydrogenase
GulLDH: L-gulono-1,4-lactone dehydrogenase
GulLO: L-gulono-1,4-lactone oxidase
GUO: D-gluconolactone oxidase
L-GalL: L-galactono-1,4-lactone
L-GulL: L-gulono-1,4-lactone
SEC: size exclusion chromatography.
