# Supplementary figures and images for: Characterization of a L-Gulono-1,4-Lactone Oxidase Like Protein in the Floral Nectar of Mucuna sempervirens, Fabaceae
Source: Front Plant Sci. 2018 Jul 30;9:1109. doi: 10.3389/fpls.2018.01109 (PMC6077269; doi:10.3389/fpls.2018.01109)

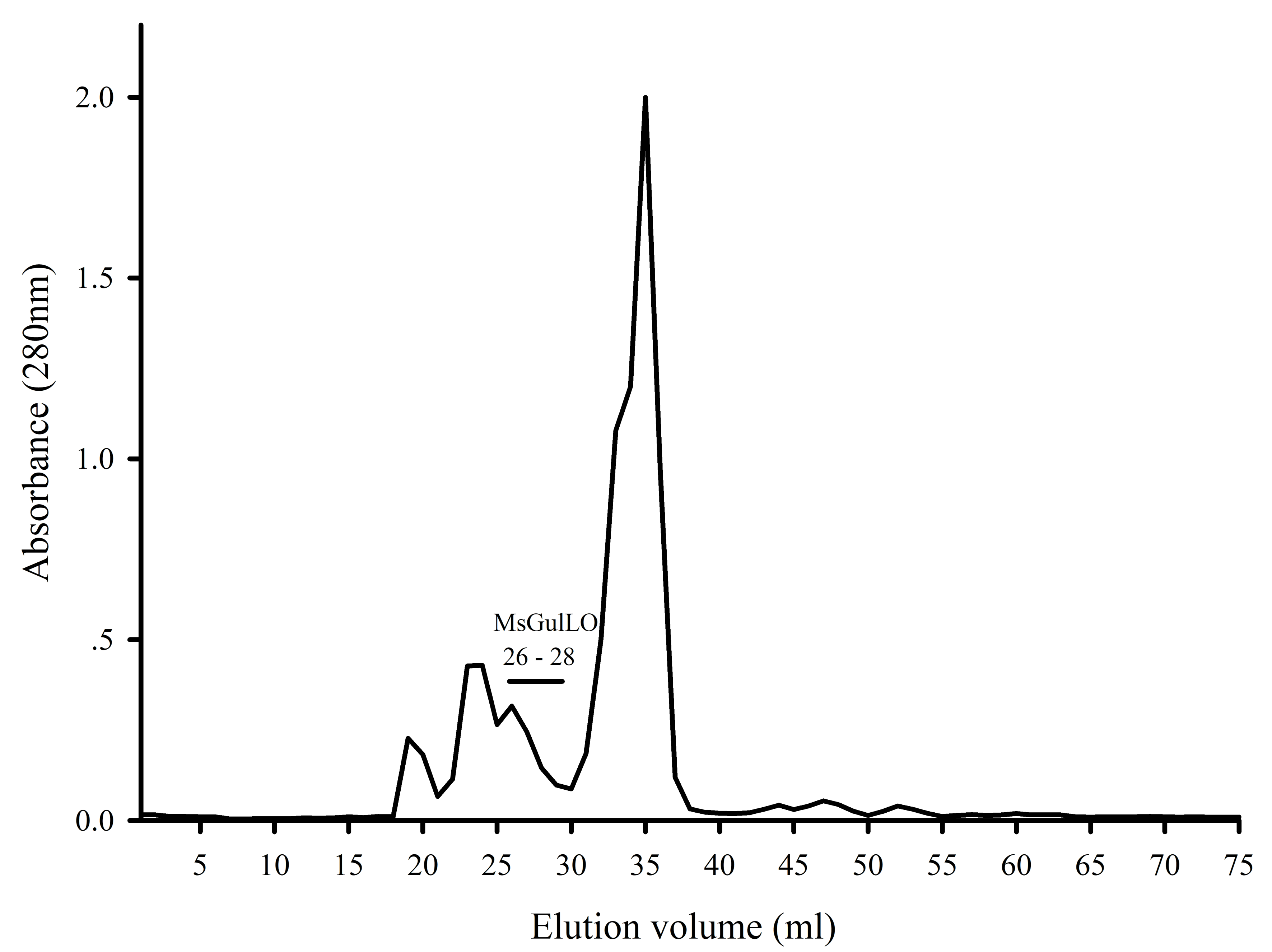

Supplement: FIGURE S1 — Elution profile of MsGulLO on a Superdex-75 size exclusion chromatography column. [file Image_1.TIF]
