# Supplementary material for: Characterization of a L-Gulono-1,4-Lactone Oxidase Like Protein in the Floral Nectar of Mucuna sempervirens, Fabaceae
Source: Front Plant Sci. 2018 Jul 30;9:1109. doi: 10.3389/fpls.2018.01109 (PMC6077269; doi:10.3389/fpls.2018.01109)
Supplement: TABLE S1 — Primers used for MsGulLO and MsGLDH cDNA cloning and qPCR. [file Table_1.DOC]

Table S1 Primers used for *MsGulLO* and *MsGLDH* cDNA cloning and qPCR

| Oligo name | Sequence (5’-3’) | | Application | |  |
| --- | --- | --- | --- | --- | --- |
| GulLO-F | GGCACTGGTGCTCATGGAAGC | | | 3’ RACE |  |
| GulLO-R1 | ATGACCCAGAAGCCTGAAGGTC | | | 5’ RACE |  |
| GulLO-R2 | CTGCAAACTCATGTTGCTGTCC | | | 5’ RACE |  |
| GulLORTF | CATCCGAACCACAGAGGATG | | | qPCR |  |
| GulLORTR | CATGACCCAGAAGCCTGAAG | | | qPCR |  |
| GLDH-F | CAAGAAGGCGCAGATCTTCC | | | 3’ RACE |  |
| GLDH-R1 | | AACTTCAGCCACAACTCCGAG | | 5’ RACE | |
| GLDH-R2 | CTTGCACCAGTGCCATGTGC | | | 5’ RACE |  |
| GLDHRTF | AGTCCTGCTTCAAGCCCATC | | | qPCR |  |
| GLDHRTR | CCTGAGTCAGATGCCTGTAG | | | qPCR |  |
| 18SF | ATTCTATGGGTGGTGGTGC | | | qPCR |  |
| 18SR | CCATCCAATCGGTAGGAGC | | | qPCR |  |
