# Supplementary material for: Characterization of a L-Gulono-1,4-Lactone Oxidase Like Protein in the Floral Nectar of Mucuna sempervirens, Fabaceae
Source: Front Plant Sci. 2018 Jul 30;9:1109. doi: 10.3389/fpls.2018.01109 (PMC6077269; doi:10.3389/fpls.2018.01109)
Supplement: TABLE S3 — MsGulLO peptides identified by mass spectrometry (MALDI-TOF/TOF). [file Table_3.DOCX]

Table S3. MsGulLO peptides identified by mass spectrometry (MALDI-TOF/TOF)

| Peptide | Mass | | Sequence | Position |
| --- | --- | --- | --- | --- |
|  | Observed | Predicted |  |  |
| 1 | 916.47 | 916.50 | VLEIDAEK | 108-115 |
| 2 | 927.42 | 927.45 | GYFCRPGK | 553-560 |
| 3 | 991.47 | 991.52 | IATNNLMAK | 322-330 |
| 4 | 992.48 | 992.50 | NFIEDVQK | 395-402 |
| 5 | 995.37 | 995.41 | MTCPWDSR | 368-375 |
| 6 | 1002.50 | 1002.54 | VVAWASQNK | 65-73 |
| 7 | 1029.50 | 1029.53 | YGGKPHWGK | 472-480 |
| 8 | 1108.50 | 1108.55 | NVAFEGVMNK | 483-492 |
| 9 | 1260.65 | 1260.69 | SLPSQELAFIR | 291-301 |
| 10 | 1441.61 | 1441.68 | SICEAEQVFYPR | 46-57 |
| 11 | 1602.68 | 1602.74 | GFCGIENYNGILMR | 410-423 |
| 12 | 1620.69 | 1620.76 | TTEDVQELISDADGK | 302-316 |
| 13 | 1648.68 | 1648.75 | QEDAIDFDITYYR | 435-447 |
| 14 | 1753.80 | 1753.89 | VHTLTEQHEYLNAAK | 193-207 |
| 15 | 1937.91 | 1938.01 | LYEDIIEEVEQLGIFK | 456-471 |
| 16 | 2177.76 | 2177.91 | EGCALEGLCICSQDNHCAPSK | 532-552 |
| 17 | 2637.15 | 2637.35 | GSSVHQHVLHLTIVTPSGPHDGYAK | 168-192 |
